# 808 nm light triggered lanthanide nanoprobes with enhanced down-shifting emission beyond 1500 nm for imaging-guided resection surgery of tumor and vascular visualization

**DOI:** 10.7150/thno.41967

**Published:** 2020-05-23

**Authors:** Youbin L i, Mingyang Jiang, Zhenluan Xue, Songjun Zeng

**Affiliations:** Synergetic Innovation Center for Quantum Effects and Application, Key Laboratory of Low-dimensional Quantum Structures and Quantum Control of Ministry of Education, Key Laboratory for Matter Microstructure and Function of Hunan Province, School of Physics and Electronics, Hunan Normal University, Changsha, 410081, P.R. China.

**Keywords:** Nd^3+^-sensitized core-shell nanoparticles, boosting NIR-II emission, eliminated overheating effect, non-invasive tumor vascular imaging, imaging-guided surgery

## Abstract

Lanthanide based nanoprobe with high efficient down-shifting second near-infrared (NIR-II, 1000-1700 nm) emission has emerged as a promising agent for tumor-associated vascular visualization. However, most of the developed lanthanide-based NIR-II-emissive probes are activated by 980 nm laser, leading to the concern of biological overheating effect. Herein, the high quality 808 nm laser activated NaYF_4_:Gd/Yb/Er/Nd/Ce@NaYF_4_:Nd core-shell nanoprobes with significantly improved NIR-II emission beyond 1500 nm and eliminated overheating effect were developed for imaging-guided resection surgery of tumor and vascular visualization.

**Methods**: The core-shell nanoprobe with boosted NIR-II emission and eliminated heating effect was achieved with combination of Nd-sensitizing and Ce-doping strategies. The NIR-II optical imaging and toxicity assessment were demonstrated by *in vivo* and *in vitro* experiments.

**Results**: The designed core-shell nanoprobe presented superior NIR-II emission beyond 1500 nm than the core only nanoparticle and NIR-II emission intensity was improved up to 11.0 times by further suppressing the upconversion (UC) pathway through doping Ce^3+^. More importantly, non-invasive tumor vascular imaging and NIR-II optical imaging-guided surgical resection of tumor were successfully achieved.

**Conclusion**: It is expected that the Nd-sensitized lanthanide-based nanoprobe with significant improvement in NIR-II emission and eliminated overheating effect is a highly promising probe for NIR-II imaging, making it more competitive in non-invasive vascular imaging and imaging-guided tumor resection surgery.

## Introduction

The complete resection of tumors has emerged as the primary effective treatment modality for malignancies 1-6. Therefore, visualization of the full margins of the tumor during the surgery is of utmost importance for the real-time surgical guidance of tumor and improving diagnostic accuracy 1,7,8. However, conventional imaging modalities such as magnetic resonance imaging (MRI), computed tomography (CT), etc. still remain a great challenge for imaging of tiny tumor, owing to the poor resolution and limited sensitivity 1,7. Compared with the existed imaging modalities, optical imaging with some unique advantages including high spatial resolution and high sensitivity, has received tremendous attention for both imaging-guided tumor resection and visualization of tumor vascular structures 9. To date, considerable efforts have been devoted to utilizing the visible (400-650 nm) and near-infrared wavelength (700-900 nm, NIR-I) emissive probe for delineating the margins of the tumor in real-time tumor resection surgery 10-19. However, the relatively large light scattering of NIR-I and visible light results in adverse effects for precisely delineating the tumor margins owing to the low spatial resolution imaging induced by the large scattering. Recently, NIR-II optical imaging is emerged as an essential technique for precisely pinpointing the tumor margins owing to its significantly reduced light scattering and low-tissue autofluorescence 20,21.

Thus far, various NIR-II emitters, such as carbon nanotubes 22-24, quantum dots (QDs) 25-27, small molecule dyes 28-38 and rare earth-based nanoparticles 39-44 have been developed for *in vivo* NIR-II biological imaging. Especially, lanthanide-based nanoprobes with efficient 1525 nm emission located in the NIR-IIb window, highly controlled particle size, low biotoxicity, high photostability, and high efficiency, are emerged as a promising agent in NIR-II based bioimaging field 41. Moghe's group 20 demonstrated the rare-earth-doped biological composites as NIR-II agent for *in vivo* multispectral bioimaging. Recently, Dai's group 39 performed a pioneering study for designing bright lanthanide based nanoprobe (NaYbF_4_:Er@NaYF_4_) *via* doping Ce^3+^ for fast brain vascular imaging under 980 nm laser excitation. And, our previous study 41 reported the Yb/Er-doped lanthanide nanoprobes for NIR-IIb optical-guided non-invasive brain vascular imaging and small tumor diagnosis. However, most of the reported lanthanide-based nanoparticles 39,41 containing sensitizer of Yb^3+^ and activator of Er^3+^ are activated by 980 nm laser to generate NIR-IIb emission, leading to an overheating effect in bio-tissues and subsequently resulting in the damage of biological tissues and cell death 45. To address this issue, shifting the excitation wavelength to 808 nm 45, 46 to overcome the overheating issues usually caused by the traditional 980 nm light is highly desirable. Nevertheless, in 808 nm laser activated lanthanide NIR-IIb emitters, a competitive process has also existed in the simultaneous down-shifting NIR-IIb and UC emitting lanthanide probes. Therefore, developing 808 nm laser activated NIR-IIb emissive nanoprobe with enhanced NIR-IIb emission, high biocompatibility, minimized heating effect, and high photostability for delineating the tumor margins and visualizing tumor vascular structure is urgently required.

Herein, we have explored the Nd-sensitized core-shell NaYF_4_:Yb/Er/Ce/Nd@ NaYF_4_:20%Nd nanoprobe with enhanced 1525 nm emission and high quantum yield (QY) under an 808 nm excitation for NIR-IIb bioimaging by the integration of Nd-sensitizing and Ce-doping strategies. Significantly, doping of Nd^3+^ in the shell is acted as a sensitizer to harvest pump photons at 808 nm, which solves the 980 nm laser induced overheating issue. The nanoparticles were converted to an aqueous phase by using polyacrylic acid (PAA) modification. The PAA-modified Nd-sensitized core-shell (PAA-C/S) nanoparticles with high QY, high biocompatiblity and bright 1525 nm emission hold great promising for high-resolution tumor vascular imaging/resection surgery without the concern of overheating effect under the 808 nm laser excitation.

## Results and Discussion

### Fabrication and characterization of Nd-sensitized core-shell nanoparticles

**Scheme 1:**
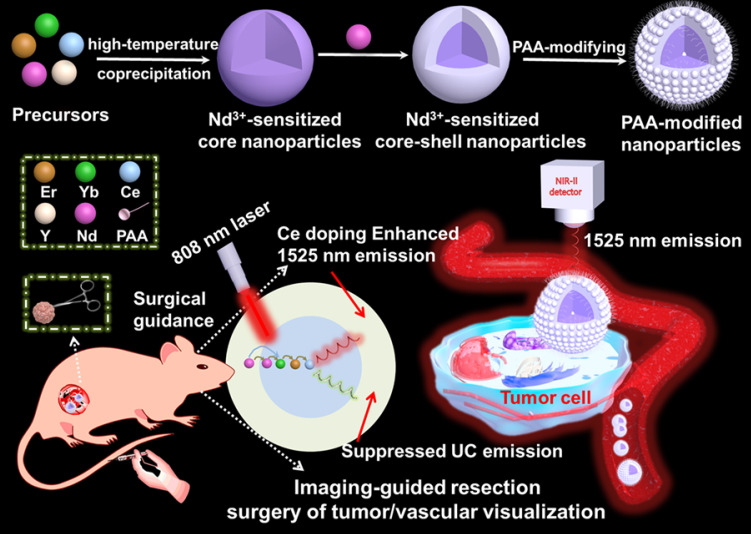
Schematic illustration of the Nd^3+^-sensitized core-shell nanoparticles with enhanced 1525 nm emission for NIR-IIb optical imaging-guided resection surgery of tumor and vascular visualization.

**Figure 1:**
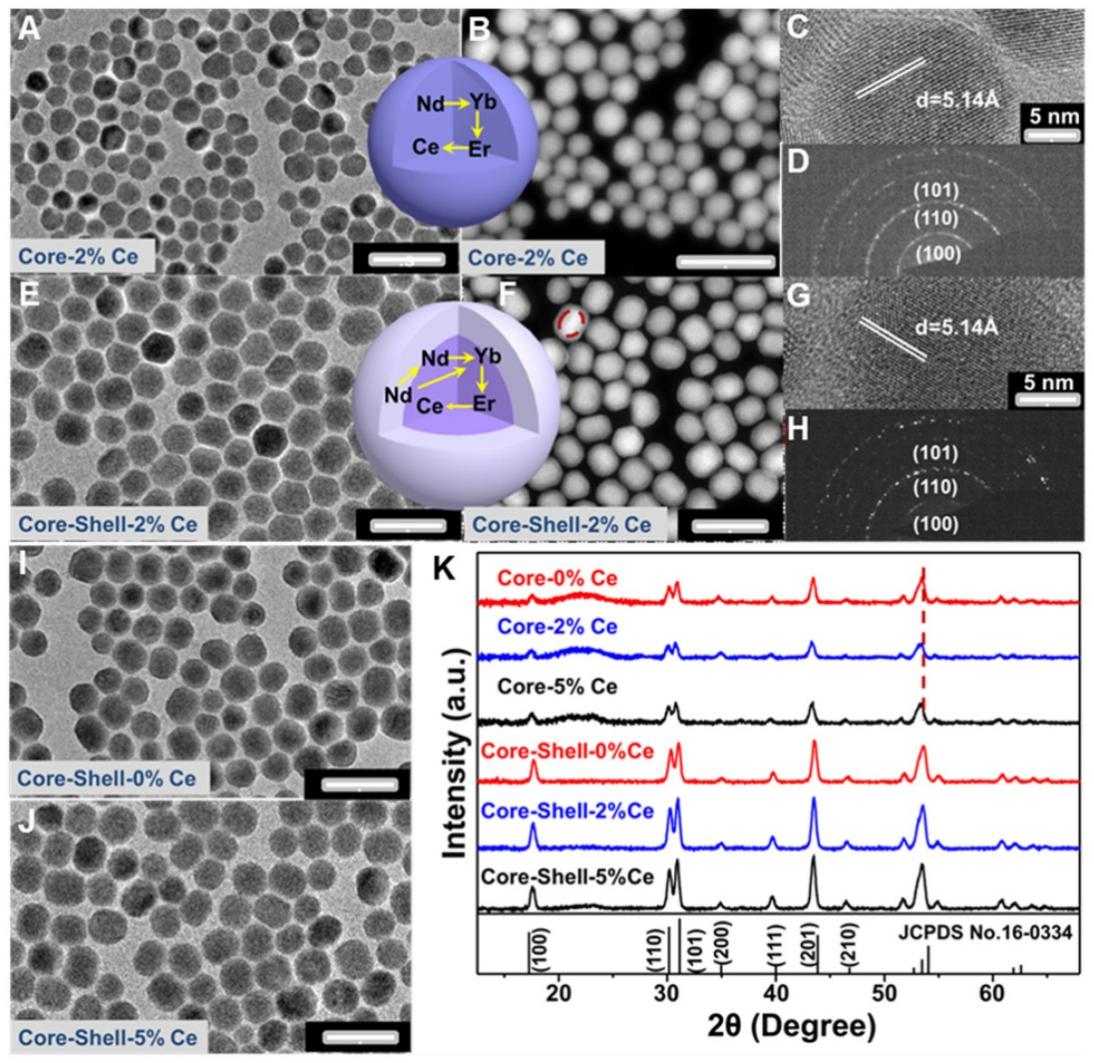
(**A-D**) TEM, STEM, HRTEM and SAED images of NaYF_4_:40%Gd/20%Yb/ 2%Ce/2%Er/1% Nd core only nanoparticles, respectively. (**E-H**) TEM, STEM, HRTEM and SAED images of NaYF_4_:40%Gd/20%Yb/2%Ce/2%Er/1%Nd@NaYF_4_:20%Nd core-shell nanoparticles, respectively. (**I**) and (**J**) TEM images of the 0 mol% and 5 mol% Ce doped NaYF_4_:40%Gd/20%Yb/2%Er/1%Nd@NaYF_4_:20%Nd core-shell nanoparticles, respectively. (**K**) The XRD patterns of the as-prepared core and core-shell nanoparticles.

The Nd-sensitized NaYF_4_:Gd/Yb/Er/x%Ce/Nd@NaYF_4_:Nd core-shell nanoparticles doped with different concentrations of Ce^3+^ in the core were synthesized by a modified layer-by-layer high-temperature co-precipitation method (**Scheme 1**) 45,47. The crystal structure and phase of the as-prepared core and core-shell nanoparticles were studied by using the transmission electron microscopy (TEM) and X-ray powder diffraction (XRD). As illustrated in **Figure 1**, the typical TEM image (**Figure 1A**) and the corresponding scanning TEM (STEM) image (**Figure 1B**) of the as-prepared NaYF_4_:40%Gd/20%Yb/2%Er/2%Ce/1%Nd core nanoparticles showed spherical and highly uniform structure. High-resolution TEM (HRTEM) image of a single nanoparticle taken from **Figure 1A** further reveals the lattice fringe with a d-spacing of 5.14 Å (**Figure 1C**), which is in good agreement with the (100) crystal plane of the hexagonal phase NaYF_4_ 48. Moreover, the selected area electron diffraction (SAED) results of the core nanoparticles (**Figure 1D**) also indicate the formation of the hexagonal phase structure. For comparison, different concentrations of Ce^3+^ doped core nanoparticles were further synthesized, demonstrating the similar lattice fringes and hexagonal phase structure (**Figure S1A**-**1F**). Then, to improve the emission intensity, a shell of NaYF_4_:20%Nd was further precipitated on the core sample *via* eliminating the surface quenching effect. The TEM (**Figure 1E**, **1I**, **1J**) results of the core-shell nanoparticles showed a larger size than the core nanoparticles, indicating the successful fabrication of core-shell nanoparticles with hexagonal phase structure (**Figure 1G** and **1H**) 46. Moreover, STEM image (**Figure 1F**) further demonstrated the different contrast effect between the core and shell (marked by the red line in **Figure 1F**), verifying the core-shell structure. Energy dispersive X-ray spectroscopy (EDS) analysis of the core-shell nanoparticles with doping 2% Ce^3+^ in core further confirmed the presence of Na, Y, F, Gd, Er, Nd and Ce elements (**Figure S1G**). The XRD patterns (**Figure 1K**) of the as-prepared core and core-shell nanoparticles are indexed exactly as the pure hexagonal phase structure (JCPDS: 16-0334) without Ce^3+^ doping. And, by increasing the contents of Ce^3+^ ions from 2% to 5 mol%, no extra diffraction peaks were observed, further revealing the formation of hexagonal phase structure for both core and core-shell nanoparticles. In addition, the diffraction peaks gradually shift to the lower diffraction angle with increasing the doped content of Ce^3+^, owing to the substitution of Y^3+^ (r = 1.159 Å) 49 by larger Ce^3+^ (1.283 Å) 49. These results further demonstrate the successful incorporation of Ce^3+^ into the host matrix.

### Remarkable enhancement of NIR-IIb emission in Nd-Sensitized core/shell nanoparticles

**Figure 2:**
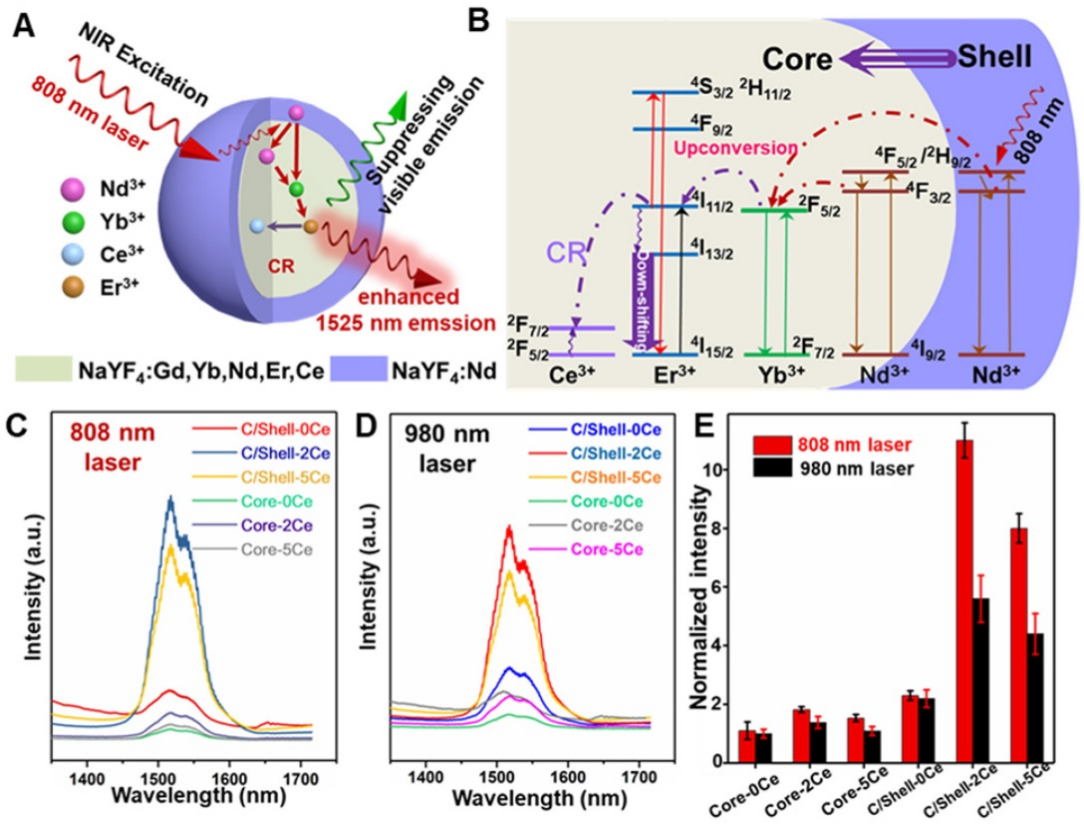
(**A**) Schematic diagram of the enhanced down-shifting emission in Nd-sensitized core-shell system under 808 nm excitation. (**B**) The energy transfer mechanism from Nd to Yb/Er. NIR-IIb emission spectra of the Nd-sensitized system under (**C**) 808 nm laser and (**D**) 980 nm laser excitation. (**E**) The down-shifting emission intensity of the core and core-shell nanoparticles doped with difference Ce^3+^ under 980 and 808 nm laser excitation.

To address the overheating effect induced by 980 nm laser and suppress the UC emission, a rational route with a combination of Nd-sensitized and Ce doping was explored. As demonstrated in **Figure 2A**, the Nd^3+^ was introduced in the core and shell as the alternative sensitizer to Yb^3+^. The Nd^3+^ holds an excellent absorption band around 800 nm, leading to the efficient energy transfer from Nd^3+^ to Yb^3+^ 46 and shifting the excitation wavelength from 980 nm to 808 nm. To reveal this, the UC and down-shifting emission spectra of the Nd^3+^-sensitized core and core-shell nanoparticles were studied. As shown in **Figure 2**, **Figure S2** and **Figure S3**, multiple emissions of Er^3+^ spanning from the UC emitting band at 525/545 nm and 650 nm to the down-shifting emitting band at 1525 nm were observed under 808 nm laser excitation. Notably, in comparison with the 980 nm light excitation, the Nd-sensitized core-shell nanoparticles exhibit brighter down-shifting emission under 808 nm irradiation (**Figure 2E**), indicating the enhancement in emission intensity. **Figure 2B** presents the mechanism of the UC/down-shifting emission processes. As demonstrated, the ^4^F_5/2_ state of Nd^3+^ was first transferred to the ^4^F_3/2_ state through non-radiative relaxation after 808 nm excitation 45,46. The photon energy was then absorbed by the adjacent Yb^3+^ in the core through energy transfer between Nd and Yb, resulting in the population of the ^2^F_5/2_ state of Yb^3+^ 45. Then the Yb^3+^ will act as the energy translation ion to transfer the energy to the activator ions of Er^3+^ in the core 45. And then, the UC emission of Er^3+^ can be ascribed to the transition of ^4^I_11/2_ state to the higher ^2^H_11/2_ and ^4^S_3/2_ energy level via further absorbing the excitation energy from Nd^3+^, while the down-shifting emission is attributed to the rapid phonon mediated non-radiative decay from ^4^I_11/2_ to^ 4^I_13/2_ level 39. It should be noted that both the UC and down-shifting emissions are highly dependent on the electronic level of ^4^I_11/2_, subsequently leading to the strong competitive process between the UC and down-shifting emissions 39,50-53. Therefore, it was urgently desired to further improve the down-shifting emission intensity by suppressing the UC emission in the Nd-sensitized system.

Fortunately, the energy level gap between ^2^F_5/2_ and ^2^F_7/2_ states of Ce^3+^ is about 2300 cm^-1^, which shows a small mismatch with the energy level gap between ^4^I_11/2_ and ^4^I_13/2_ state (about 3700 cm^-1^) of Er^3+^. Therefore, doping Ce^3+^ can favor the population of the ^4^I_13/2_ level of Er^3+^, through the cross-relaxation (CR, **Figure 2B**) between Er^3+^ (^4^I_11/2_→^4^I_13/2_) and Ce^3+^ (^2^F_5/2_→^2^F_7/2_), finally enhancing the down-shifting 1525 nm emission of Er^3+^ 39. Thus, a general Ce^3+^ doping strategy was further introduced to boost the down-shifting 1525 nm emission *via* suppressing the UC emission pathway in our designed Nd-sensitized system by using 808 nm laser as the excitation source. As shown in **Figure 2C-2E** and **Figure S3**, in comparison with the Ce-free core nanoparticles, when doping 2 mol% Ce^3+^, significant enhancement in 1525 nm emission (~ 11 folders) with suppressing UC emissions was achieved. And the down-shifting emission tended to decrease at a higher Ce^3+^ doping content, which was mainly attributed to the concentration quenching effect. The concentration quenching usually occurs through dipole-dipole interaction between the lanthanide ions, which is highly dependent on R^-6^ (R indicates the inter-ionic distance between the rare-earth ions) 53. Thus, increasing the doped Ce contents may decrease the inter-ionic distance, resulting in the concentration quenching phenomenon, which is also consistent with the previous report 39.

For further biomedical application, the hydrophobic NaYF_4_:Yb/Gd/2mol% Ce/Er/Nd@NaYF_4_:Nd core-shell nanoparticles were first converted into the aqueous phase by using a PAA-modifying method 54. The photostability of the PAA modified core-shell (denoted as PAA-C/S) nanoparticles was further studied in water, phosphate buffer solution (PBS) and fetal bovine serum (FBS) solutions under continuous 808 nm laser irradiation for 2 h. As shown in **Figure S4** and **S5**, high photostability was observed. The QY of the down-shifting emission of the 2 mol% Ce doped PAA-C/S nanoparticles in water was measured by using a standard IR-26 dye (QY = 0.5%, dissolved in 1,2-dichloroethane) as the reference 39. The QY is determined to be about 2.9% in water. *In vitro* phantom bioimaging (**Figure S6**) of the PAA-C/S nanoparticles in water was further tested, also validating the improved 1525 nm emission under 808 nm laser excitation than 980 nm laser excitation, which was consistent with the aforementioned emission spectra. Therefore, it is expected that the explored PAA-C/S nanoparticles with high photostability, high QY, and bright 1525 nm emission are ideal probes for NIR-IIb optical-guided bioimaging application.

### Elimination of overheating effect *via* Nd-sensitization

**Figure 3:**
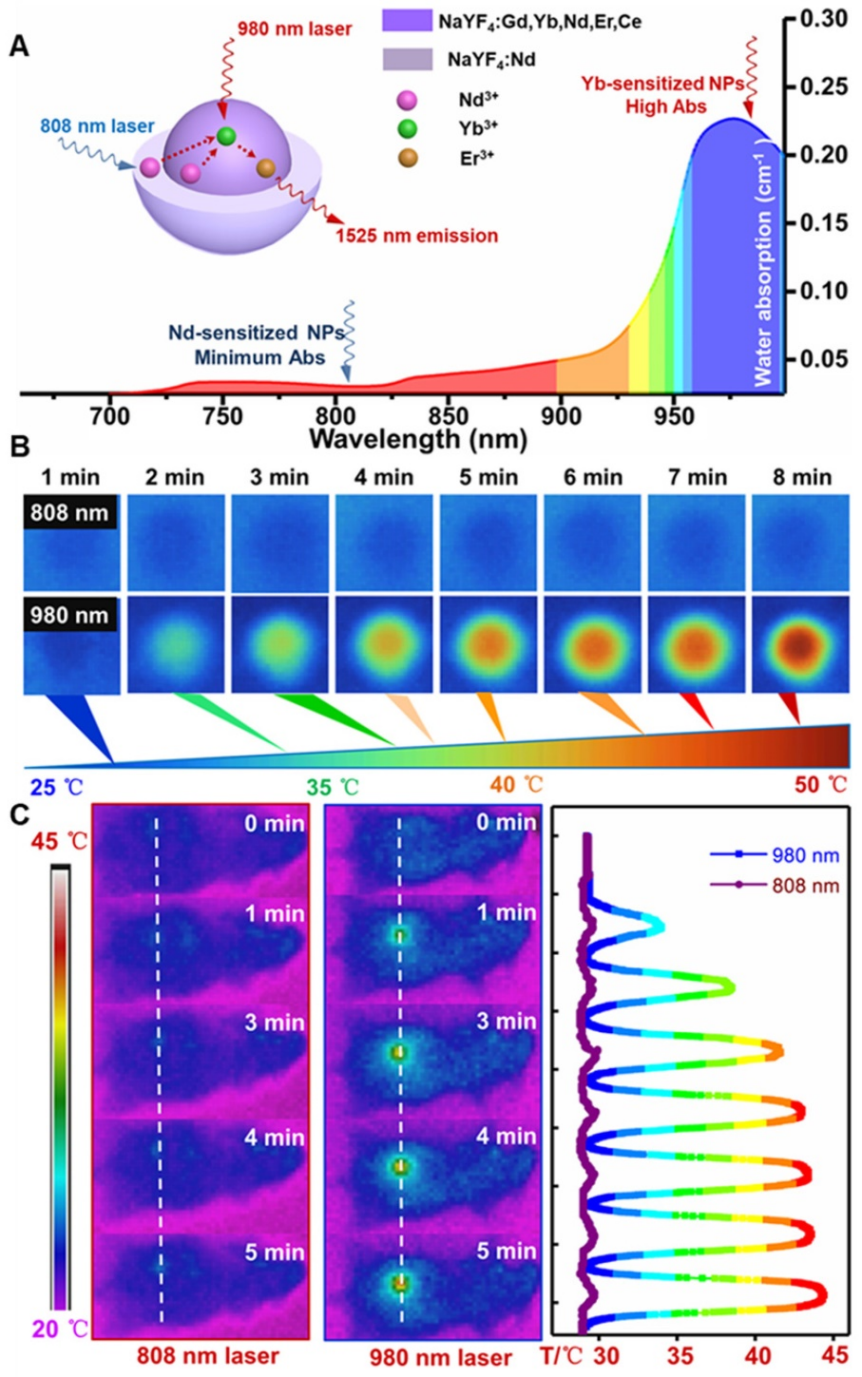
(**A**) The absorption spectra of water in the NIR region. (**B**) Infrared thermal images of PAA-C/S nanoparticles in water under continuous 808/980 nm laser irradiation for 8 min. (**C**) *In vivo* infrared thermal images of the nude mice under continuous 980/808 nm laser irradiation and the corresponding temperature variation profiles along the dotted white lines.

For further evaluating the laser induced overheating problem, the optical absorption spectra of water were first tested. As shown in **Figure 3A**, the absorption coefficient of water at 808 nm is lower than the value at 980 nm. Therefore, laser irradiation induced overheating effect on biological tissues can be greatly minimized by using an 808 nm light source 45. Thus, the thermal effect of the PAA-C/S solutions under continuous 980/808 nm laser irradiation was studied. As shown in **Figure 3B**, the PAA-C/S solution exhibits an unobvious heating effect after continuous 808 nm laser irradiation for 8 min. In contrast, the temperature increased from 27 °C to nearly 50 °C under 980 nm laser irradiation with power density of 1 W/cm^2^, verifying the eliminated overheating effect by using the alternative 808 nm laser irradiation. To further reveal the eliminated overheating effect, *in vivo* heating effects were investigated. As shown in **Figure 3C** and **Figure S7**, upon irradiation with 980 nm laser at low power (0.15 and 0.3 W/cm^2^), the temperature of the irradiation area showed a limited increase after 7 min irradiation. An obvious local heating effect with temperature increment up to 20 °C was observed under continuous 980 nm laser irradiation for 7 min (0.6 W/cm^2^) and 5 min (1 W/cm^2^). In contrast, only a slight increase in temperature (~ 2 °C) was recorded under the 808 nm laser irradiation for 7 min at 1 W/cm^2^. These results revealed that Nd-sensitized core/shell nanoprobes with a minimized heating effect under 808 nm laser irradiation were more beneficial for *in vivo* biomedical application.

### Non-invasive NIR-IIb optical-guided tumor vascular detection

**Figure 4:**
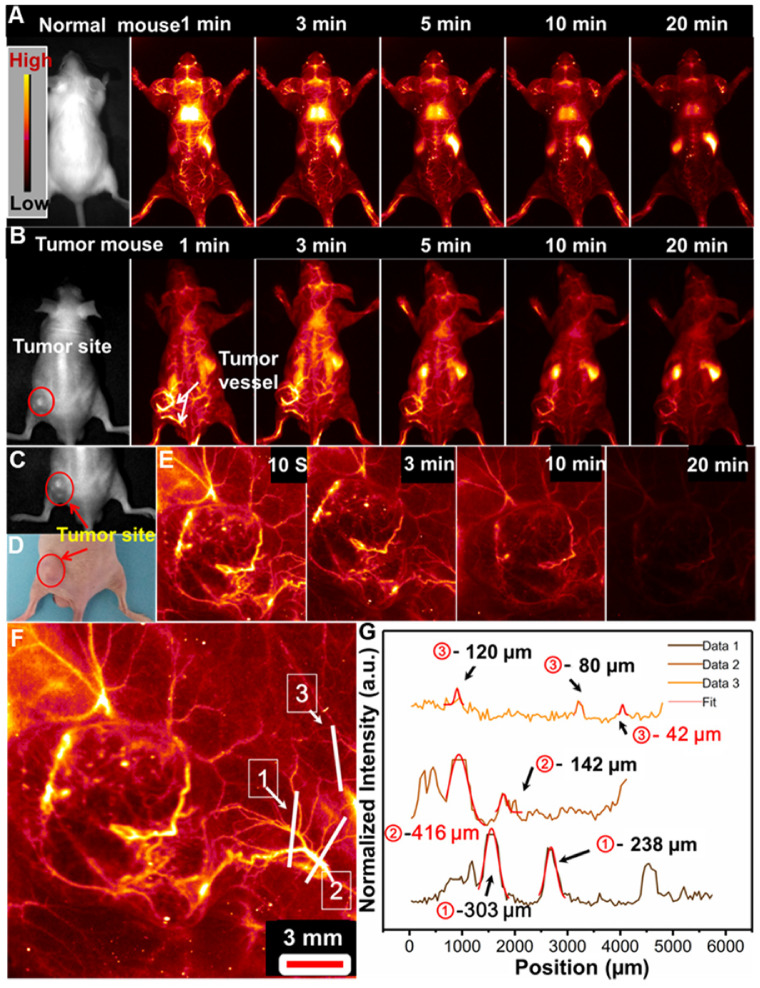
*In vivo* whole-body NIR-IIb optical-guided vessel imaging of the (**A**) normal mouse and (**B**) lung tumor bearing mouse after intravenous injection of the PAA-C/S solutions under 808 nm laser excitation. (**C**) A bright field image and (**D**) digital photograph of the lung tumor-bearing mouse. (**E**) Time coursed NIR-IIb vessel imaging of the lung tumor bearing mouse under the 808 nm laser excitation (FOV of 26 mm×21 mm). (**F**) A magnified tumor vascular image. (**G**) The cross-sectional fluorescence intensity profiles along the white lines 1, 2 and 3 marked in (**F**).

Tumor formation and growth are highly dependent on the blood vessels around the tumor region 55,56. And highly sensitive detection of tumor associated vessels may provide more information on the tumor growth and leakage behavior in tumor region 55,56. Before *in vivo* application, the cell toxicity of the PAA-C/S nanoprobe was evaluated by using the 3-(4,5-dimethylthiazol-2-yl)-2,5 diphenyl-tetrazolium bromide (MTT) assays. As demonstrated in **Figure S8,** the cell viability is measured to be about 90% when treated with 0-1 mg/mL PAA-C/S nanoprobes, indicating the high biocompatibility of the probes for *in vivo* bioimaging application. To further evaluate the feasibility of the PAA-C/S nanoprobes for tumor vascular imaging, NIR-IIb optical-guided vascular imaging of the normal mouse and lung tumor-bearing nude mouse was performed. As shown in **Figure 4A**, bright fluorescence signals in the normal mouse vessels were clearly observed after 1 min injection. The vascular signal was decreased as prolonging time and almost disappeared after 20 min injection, suggesting the efficient *in vivo* vascular imaging. Then the tumor vascular imaging by using the lung tumor-bearing nude mouse was performed. As shown in **Figure 4B**, after 1 min injection, the PAA-C/S nanoprobes were first entered into the systemic blood circulation, abundant vessels in and around the tumor site were clearly observed. And the fluorescence signal in the tumor vessels could still be clearly distinguished after 20 min injection, validating the feasibility of PAA-C/S nanoprobes for a long time and highly sensitive tumor vascular imaging. As time elapsed, the signals of the whole body/tumor blood vessels were almost vanished after 20 min injection, and the PAA-C/S nanoprobes were gradually entered into the liver, spleen and inner tumor region, leading to the enhanced fluorescence intensity in liver and spleen.

We further performed the high magnification NIR-IIb optical-guided lung tumor vascular imaging with the field of view (FOV) of 26 mm×21 mm. As shown in **Figure 4C-4E**, the tumor vessels were immediately identified at 10 s post-injection of PAA-C/S solutions at the tail vein. The fluorescence signal of the tumor vessels was gradually attenuated after 7 min injection and almost vanished after 20 min injection. Then the cross-sectional intensity profiles of the tumor vessels marked by the white lines were analyzed by using the Gaussian fitted method. An ultrasmall tumor vessel with a diameter of 42 µm (**Figure 4F** and **4G**) was clearly observed, which was close to the limit of resolution of 41 µm at the current imaging condition. Therefore, with the high sensitivity and high spatial resolution tumor vessel imaging, the designed Nd-sensitized PAA-C/S probes can be used as promising agents for tumor associated vasculature diagnosis.

To further reveal the tumor vessel imaging, another colorectal tumor-bearing nude mouse was intravenously injected with PAA-C/S solution for imaging. As shown in **Figure S9A**, a lot of vessels in the tumor site were also visualized, which was similar to the aforementioned results. Then, the fluorescence intensity profiles along the marked white lines were evaluated. As presented in **Figure S9B**-**S9E**, the tumor vessels with fitted diameter from 62 to 162 μm were observed. Then the colorectal tumor bearing mouse was sacrificed after 24 h injection for *ex vivo* NIR-IIb bioimaging. As shown in **Figure S10**, the isolated tumor exhibited a bright NIR-IIb signal, revealing the efficient accumulation of PAA-C/S nanoprobes in the tumor site based on the enhanced permeability and retention (EPR) effect 57,58. These results further validate the feasibility of tumor vascular imaging by using the explored PAA-C/S nanoparticles.

### NIR-IIb Optical Imaging-Guided Surgical Guidance

**Figure 5:**
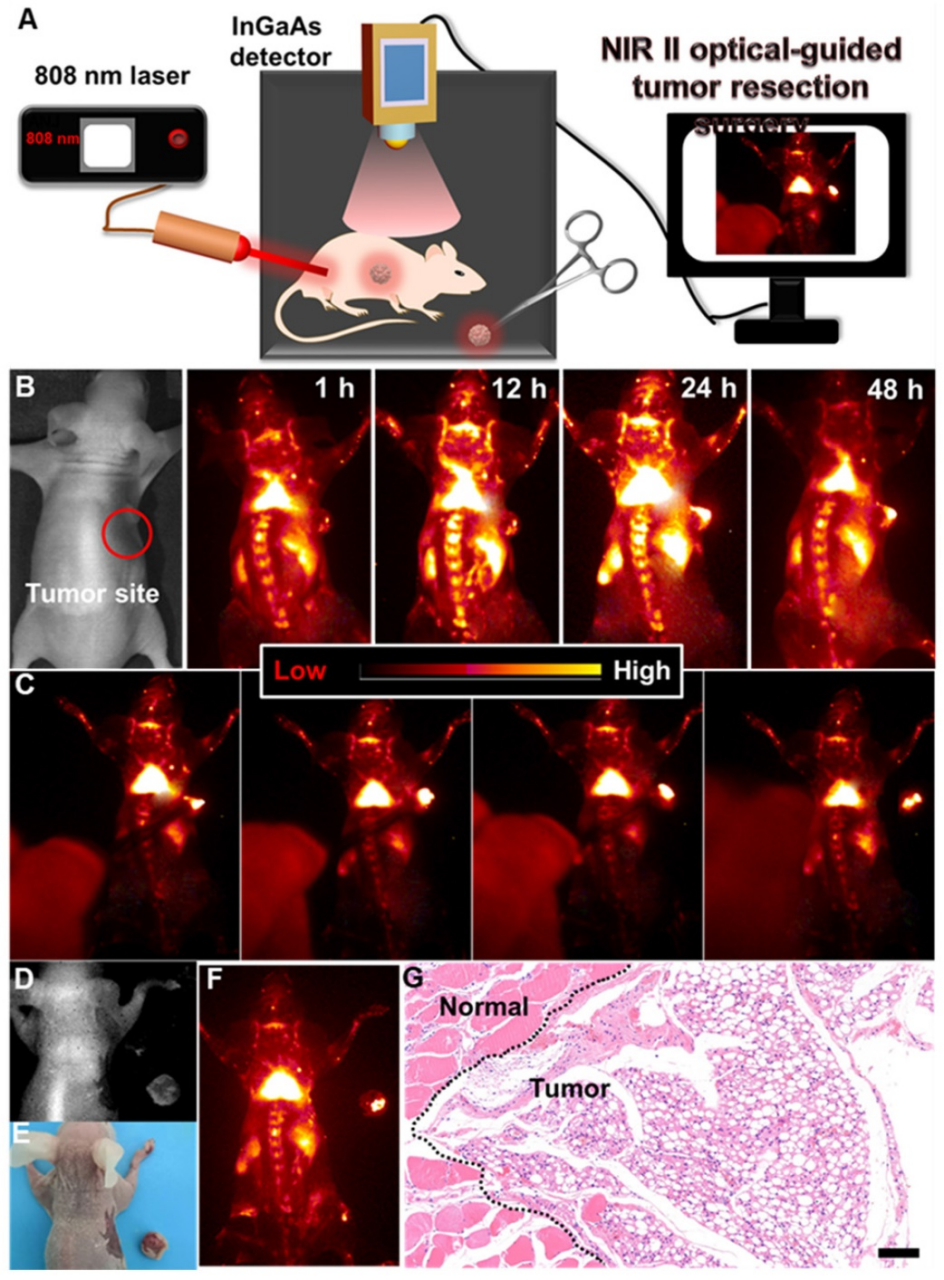
(**A**) Schematic illustration of the NIR-IIb optical imaging-guided tumor resection. (**B**) NIR-IIb bioimaging of the colorectal tumor-bearing mouse after intravenously injected with PAA-C/S nanoprobes. (**C**) Representing images of the dynamic NIR-IIb optical imaging-guided resection of tumor. (**D**) A bright field image, (**E**) digital photograph and (**F**) NIR-IIb imaging of the colorectal tumor-bearing mouse and the resected tumor. (**G**) H&E analysis of the resected tumor, the margin of the normal tissue and tumor was marked by the black dashed line.

Surgical removal of the tumor plays a crucial role in the complete cure of the tumor. And, delineating the tumor margin is highly important for precise tumor resection. Therefore, developing a highly sensitive optical probe for precise delineation of the tumor margin is significant for accurate and complete resection of the tumor. To further reveal this, NIR-IIb optical imaging-guided tumor resection (**Figure 5A**) was performed. As demonstrated, the colorectal tumor bearing nude mouse was first intravenously injected with PAA-C/S solutions. And then, slight NIR-IIb fluorescence signals (**Figure 5B**, **Figure S11**) in the tumor site were observed after 1 h injection, and the full delineation of the tumor margin can be easily distinguished after 24 h injection, indicating the effective retention of PAA-C/S nanoprobes in the tumor site. The NIR-IIb fluorescence signal slightly decreases after 48 h injection, but the complete tumor margin can still be clearly visualized, revealing the long-term retention of the PAA-C/S nanoprobes in the tumor site. The tumor to normal (T/N) tissue ratio was further investigated to confirm the accurate surgical time window. As presented in **Figure S12**, the T/N ratio of the mouse treated with PAA-C/S nanoprobe increased up to 14 at 24 h post treatment and slightly decreased to 13 after 48 h injection, which was high enough for a long time (~ 24 h) NIR-IIb optical imaging-guided tumor resection surgery. Subsequently, dynamic NIR-IIb optical imaging-guided tumor resection was demonstrated. As shown in **Figure 5C-5F**, the tumor was entirely removed from the mouse and bright NIR-IIb fluorescence signals were observed in the isolated tumor. To further reveal the complete resection, the hematoxylin and eosin (H&E) analysis was performed. As demonstrated in **Figure 5G**, the complete tumor margin can be clearly identified between the tumor and normal tissue, verifying the successful resection of tumor under the guidance of NIR-IIb optical imaging.

### *In vivo* pharmacokinetics and biodistribution evaluation

The pharmacokinetic behavior and time-dependent biodistribution of PAA-C/S nanoprobes *in vivo* were evaluated after intravenous injection. As shown in **Figure S13**, the pharmacokinetic study was evaluated by testing the NIR-IIb emission intensity of blood samples collected at different time points in a similar way to the previous report 39. The blood circulation half-life of PAA-C/S nanoprobe was measured to be about 56 min (**Figure S13B**). Besides, *ex vivo* bioimaging results indicated that the PAA-C/S nanoprobes were mainly accumulated in the liver, and spleen organs after 1 h injection (**Figure S14**), the fluorescence signals in these organs were gradually decreased after 24 h injection. By using inductively coupled plasma mass spectrometry (ICP-MS), we have further studied the quantitative distribution of the PAA-C/S nanoprobes *in vivo* by measuring the Y element content in the dissected organs at different time points (1 h, 24 h, 48 h). As demonstrated in **Figure S15**, the PAA-C/S nanoprobes were mainly accumulated in liver and spleen organs after 1 h injection and decreased after 24 h injection, which was similar to the *ex vivo* bioimaging results.

### Histological and Blood Analysis

The blood routine including white blood cell (WBC), lymph, red blood cell (RBC) and hemoglobin (HGB), and biochemistry tests including aspartate aminotransferase (AST), alanine aminotransferase (ALT), urea and creatinine were performed to evaluate the long-term retention toxicity of the probe *in vivo*. As shown in **Figure S16** and **Figure S17**, after 7 and 14 days of intravenous injection, the treated group showed no statistical difference with the control group, indicating the negligible toxicity effect of the PAA-C/S nanoprobe to liver and kidney. The histological damage was further investigated by using the H&E staining method. As presented in **Figure S18**, the main organs (heart, liver, spleen, lung, and kidney) of the normal mice after injection of PAA-C/S solutions for 15 and 30 days showed no difference with the control mouse, further indicating the high biocompatibility of the PAA-C/S nanoprobes *in vivo*.

## Conclusions

In conclusion, down-shifting NIR-IIb emissive Nd-sensitized core-shell nanoparticles with boosted 1525 nm emission and eliminated overheating effect were designed under 808 nm laser excitation. The NIR-IIb emission beyond 1500 nm can be significantly improved by 11 times with a combination of Nd-sensitizing and Ce doping method. NIR-IIb optical-guided tumor resection and associated vascular imaging with high spatial resolution were successfully achieved, owing to the high QY (2.9% in water) of the developed PAA-C/S nanoprobes. Therefore, the significant improvement in 1525 nm emission and minimum overheating effect make the developed PAA-C/S nanoprobes more competitive in non-invasive disease monitoring and imaging-guided tumor resection.

## Supplementary Material

Supplementary figures.

## Abbreviations

NIR-II: second near-infrared
UC: upconversion
QY: quantum yield
MRI: magnetic resonance imaging
CT: computed tomography
NIR-I: near-infrared wavelength
QDs: quantum dots
PAA: polyacrylic acid
PAA-C/S: PAA modified core-shell
XRD: X-ray powder diffraction
HRTEM: High-resolution TEM
SAED: selected area electron diffraction
EDS: Energy dispersive X-ray spectroscopy
STEM: Scanning transmission electron microscope
CR: cross-relaxation
PBS: phosphate buffer solution
FBS: fetal bovine serum
MTT: 3-(4,5-dimethylthiazol-2-yl)-2,5 diphenyl-tetrazolium bromide
FOV: field of view
EPR: enhanced permeability and retention
T/N: tumor to normal
H&E: hematoxylin and eosin
ICP-MS: inductively coupled plasma mass spectrometry
WBC: white blood cell
RBC: red blood cell
HGB: hemoglobin
AST: aspartate aminotransferase
ALT: alanine aminotransferase
