# Supplementary material for: 808 nm light triggered lanthanide nanoprobes with enhanced down-shifting emission beyond 1500 nm for imaging-guided resection surgery of tumor and vascular visualization
Source: Theranostics. 2020 May 23;10(15):6875–85. doi: 10.7150/thno.41967 (PMC7295047; doi:10.7150/thno.41967)
Supplement: Supplementary file 1 — Supplementary figures. [file thnov10p6875s1.pdf]

SUPPORTING INFORMATION

808 nm light triggered lanthanide nanoprobes with enhanced down-shifting emission beyond 1500 nm for imaging-guided resection surgery of tumor and vascular visualization

*Youbin Li , Mingyang Jiang , Zhenluan Xue, Songjun Zeng**

Synergetic Innovation Center for Quantum Effects and Application, Key Laboratory of Low-dimensional Quantum Structures and Quantum Control of Ministry of Education, Key Laboratory for Matter Microstructure and Function of Hunan Province, School of Physics and Electronics, Hunan Normal University, Changsha, 410081, P.R. China.

Corresponding Author: songjunz@hunnu.edu.cn.

Chemicals and materials. Rare earth $YbCl_3$•$6H_2O$ (99.99%), YCl_3•$6H_2O$ (99.99%), $ErCl_3$•$6H_2O$ (99.99%), $GdCl_3$•$6H_2O$ (99.99%), $CeCl_3$•$7H_2O$ (99.99%), $NdCl_3$ •$6H_2O$ (99.99%) were purchased from QingDa elaborate Chemical Reagent Co. Ltd (Shandong). Oleic acid (OA, 90%) and 1-Octadecene (90%), NH_4F and NaOH, CH_3OH (99.5%), anhydrous alcohol, polymers acrylic acid (PAA) and all other reagents were supplied by Sigma-Aldrich. All chemical reagents were analytical grade and used without further purification.

Synthesis of $NaYF_4$:Gd/Yb/Er/Nd/x%Ce (X=0, 2, 5) core nanoparticles. The $NaYF_4$:40Gd/18Yb/2Er/1Nd/xmol%Ce core nanoparticles were synthesized by a modified high-temperature co-precipitation method [s1]. Typically, 1 mmol of $LnCl_3$ (Ln= Yb, Y, Er, Gd, Nd and Ce with designed molar ratios), oleic acid (12 mL) and 1-octadecene (30 mL) were added into a 100 mL three-neck flask. The solution was heated at 160 ℃ for 1 h and then cooled down to 90 ℃. After that, 10 mL of methanol solution containing 0.1 g NaOH and 20 mL of methanol solution containing of 0.148 g NH_4F were added into the solution and kept stirring for 1 h at room temperature. Subsequently, the solution was heated to 305 ℃ and kept for 1 h under argon atmosphere. Finally, the solution was cooled down to room temperature. The products were precipitated with ethanol and collected by centrifugation, and finally re-dispersed in 5 mL of cyclohexane for further use.

Synthesis of $NaYF_4$:Gd/Yb/Er/Nd/x%Ce@$NaYF_4$:Nd (X=0, 2, 5) core-shell nanoparticles. In a typical procedure [s1], 0.8 mmol YCl_3 and 0.2 mmol $NdCl_3$ were added into a 100 mL three-neck flask containing oleic acid (12 mL) and 1-octadecene (30 mL). The solution was heated to 160 ℃ and kept for 1 h, and then cooled down to 90 ℃. After that, the core nanoparticles, 10 mL of methanol solution containing 0.1 g NaOH and 20 mL of methanol solution containing of 0.148 g NH_4F were added into the solution and kept stirring for 1 h at room temperature. Subsequently, the solution was heated to 305 ℃ and kept for 1 h. Finally, the solution was cooled down to room temperature. IIN

Surface modification of core-shell nanoparticles. The hydrophilic $NaYF_4$:40Gd/20Yb/2Er/1Nd/2mol%Ce@$NaYF_4$:20mol%Nd core-shell nanoparticles were prepared for further biomedical application by using a modified PAA ligand exchange method [s2]. In detail, 5 mL of the prepared core-shell nanoparticles in hexane were added into 5 mL of $NOBF_4$ (0.01M) solution in dimethylformamide (DMF). After gently shaking for 1 h, the nanoparticles were collected *via* centrifugation. Subsequently, the precipitated nanoparticles were re-dispersed in 5 mL DMF, 10 mL toluene and re-collected by centrifugation.

After that, the precipitated nanoparticles were re-dispersed in 5 mL of DMF to form a stable colloidal dispersion. 200 mg PAA was then added into the solution and heated at 80 °C for 30 min, the solution was then precipitated by addition of acetone, and washed with water. Finally, the precipitates were re-dispersed in 5 mL of PBS solution for further use.

Characterization. The morphology of the samples was characterized by TEM (FEI Tecnai F20) equipped with the energy dispersive X-ray spectroscopy (EDS, Oxford Instrument) system using an accelerating voltage of 200 kV. The X-ray diffraction (XRD) patterns were performed on a Rigaku D/max 2500 system. The upconversion luminescence spectra were recorded on a Zolix spectrophotometer (fluoroSENS 9000A) system at room temperature. The down-shifting luminescence spectra were tested by using a NIR-II spectroscopy (NIRQuest512, Ocean Optics) at room temperature. The UV-Vis absorption spectrum of the nanoparticles was measured by using a spectrophotometer system (UV-1800, Hunan Sino-Jewell Electronics Co., Ltd.). The temperature and corresponding infrared thermal images were recorded by using a Ti 95 thermal IR cameral (Ti 95, Fluke, USA).

Animal models. 8×10^{6} lung cells and colorectal cells were subcutaneously injected into the BALB/C nude mice. The nude mice were then cultured to obtain the lung and colorectal tumor model with average size of 1 cm in the original injected site for further use. The animals used in the experiments were complied with the institutional animal use of Hunan Normal University approved by the Laboratory Animal Center of Hunan Province.

NIR-IIb optical-guided blood vessel imaging and *ex vivo* bioimaging. NIR-IIb optical-guided dynamic blood vascular imaging of the normal Kunming mice and lung tumor-bearing nude mice was demonstrated by using the NIR-IIb bioimaging system [s3] (In Vivo Master, Wuhan Grand-imaging Technology Co., LTD) equipped with a thermoelectric cooled InGaAs camera (Model: NIRvana™ Camera System, operating temperature: -80 ℃, Princeton Instruments) coupled with an 808 nm diode laser as the light source. The mice were first intravenously injected with 200 μL of PAA-C/S solutions (3 mg/mL), and then we performed the blood vascular imaging (Whole body: field of view (FOV): 14.6 cm×18.3 cm; High magnification tumor vascular dynamic imaging: FOV: 26 mm×21 mm, 640×512 pixels, 41 μm/pixel) under 808 nm laser excitation (power density: 100 mW/cm^2, exposure time: 1000 ms). *Ex-vivo* bioimaging of the colorectal tumor-bearing

nude mouse was demonstrated by using the same system under identical conditions.

Quantum yield measurement. The fluorescence QY of the Nd-sensitized core-shell nanoparticles in water was measured in a similar way to the previous reports [s4, s5] by using IR-26 (dissolved in Dichloroethane (DCE), QY=0.5%) as reference. The QY was calculated by using the followed equation:

$$\Phi_s = \Phi_r (F_s/F_r)*(A_r/A_s)*(n^2_s/n^2_r)$$

where Φ represents QY, F represents the integrated photoluminescence emission intensity, A is the absorbance at the maximum excitation wavelength (808 nm) and n represents the refractive index of the solvent (n= 1.33 for water, n = 1.44 for DCE, respectively). The subscripts s and r represent the sample and reference sample, respectively. The absorbance values of the 2% Ce doped PAA-C/S nanoprobes in water and IR-26 in DCE were tested by using the spectrophotometer system. The fluorescence intensity of the above-mentioned PAA-C/S nanoprobes in water and IR-26 in DCE were tested by using the NIR-IIb bioimaging system under 808 nm laser excitation, exposure time: 500 ms, power density: 50 mW/cm^2.

***In vivo* pharmacokinetics and biodistribution evaluation.** The Kunming mice after intravenous injection of PAA-C/S solution (200 μL, 3 mg/mL) were used for pharmacokinetics and biodistribution studies. The blood half-life was measured based on the blood fluorescence intensity according to the previous report [s5]. The blood samples were collected at different time points from 1 min to 36 h. The concentrations of PAA-C/S nanoprobes in the blood were calculated based on the NIR-IIb emission intensity under 808 nm laser excitation. The biodistribution of the PAA-C/S nanoprobes in mice was also investigated by sacrificing the mice after 1 h and 24 h injection. The main organs (including the heart, liver, spleen, lung, kidney) were collected for *ex vivo* NIR-IIb bioimaging by using the same imaging system.

NIR-IIb optical-guided tumor resection. The colorectal tumor-bearing nude mice were intravenously injected with PAA-C/S solutions (200 μL, 3 mg/mL). The tumors can be clearly distinguished from the normal tissues after 24 h post-injection. Then the tumors were resected from the nude mice under real-time NIR-IIb optical bioimaging guidance and analyzed by (hematoxylin and eosin) H&E staining.

Cytotoxicity Assay. *In vitro* viability of PAA-C/S nanoprobe in 4T1 cells by using the 3-(4,5-dimethylthiazol-2-yl)-2,5 diphenyl-tetrazolium bromide (MTT) assays. Cells in a 96-well microplate (6000 cells per well) were kept at 37 °C under 5% CO_2 for 3h. Then the cell culture medium in each well was

replaced by Dulbecco's Modified Eagle Medium (DMEM) solution including 10% fetal bovine serum, 1% penicillin and streptomycin. Then the PAA-C/S nanoprobes with different concentrations (0, 100, 200, 500, 800, 1000 μg/mL) were added into the cells at 37 ℃ and with 5% CO_2 for another 24 h. Finally, the cell viability was evaluated by using a typical MTT assay.

Histology analysis. For histology analysis, the Kunming mice injected with 200 μL of PAA-C/S solutions (3 mg/mL) through tail vein after 15 days, 30 days and control group were dissected. The obtained heart, liver, spleen, lung, and kidney were isolated and stained with H&E for further detection.

Blood Analysis. Blood routine including white blood cell (WBC), lymph, red blood cell (RBC) and hemoglobin (HGB), and biochemistry tests including aspartate aminotransferase (AST), alanine aminotransferase (ALT), urea and creatinine were performed. The bloods of the Kunming mice injected with 200 μL of PAA-C/S solutions (3 mg/mL) through tail vein after 7 days, 14 days and control group were obtained for further blood analysis.

Statistical analysis. All the data were presented as the mean ± SD. The significance of the difference was determined through one-way analysis of variance (***$p < 0.001$).

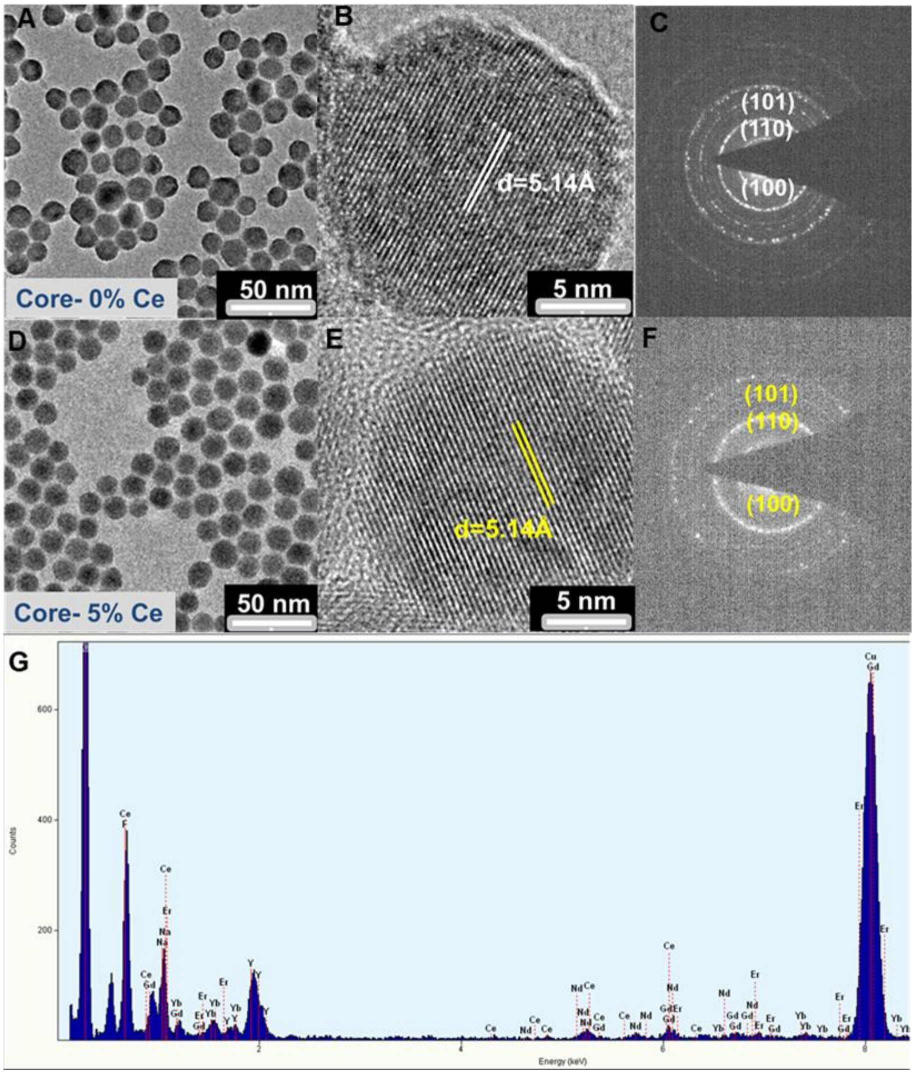

Figure S1. (A)-(C) TEM, HRTEM and SAED images of the $NaYF_4$: 40 mol%Gd/20 mol%Y/2 mol%Er/1 mol% Nd nanoparticles, respectively. (D)-(F) TEM, HRTEM and SAED images of the $NaYF_4$: 40 mol%Gd/20 mol%Yb/5 mol%Ce/2 mol%Er/1 mol% Nd nanoparticles, respectively. (G) EDS pattern of the as prepared $NaYF_4$: Gd/Yb/ /2mol%Ce/Er/Nd@$NaYF_4$:Nd nanoparticles taken from Figure 1E.

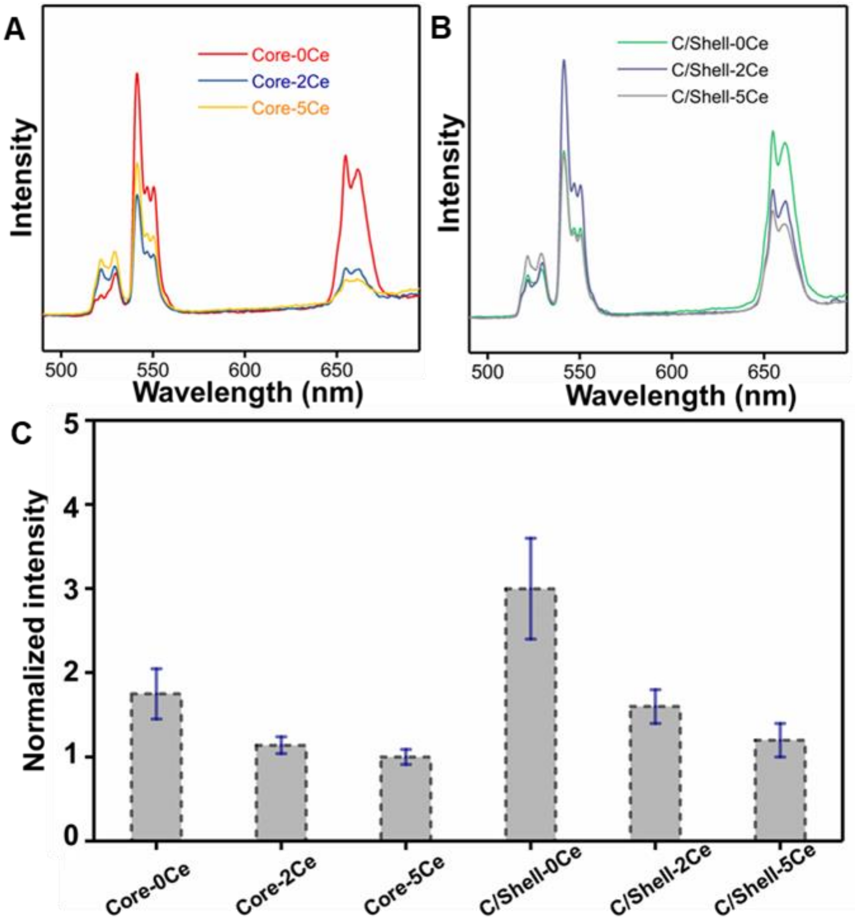

Figure S2. Upconversion emission spectra of the as-prepared Nd-sensitized (A) core and (B) core-shell nanoparticles under 980 nm laser excitation. (C) The normalized upconversion emission intensity of the difference Ce^{3+} doped core and core-shell nanoparticles under 980 laser excitation.

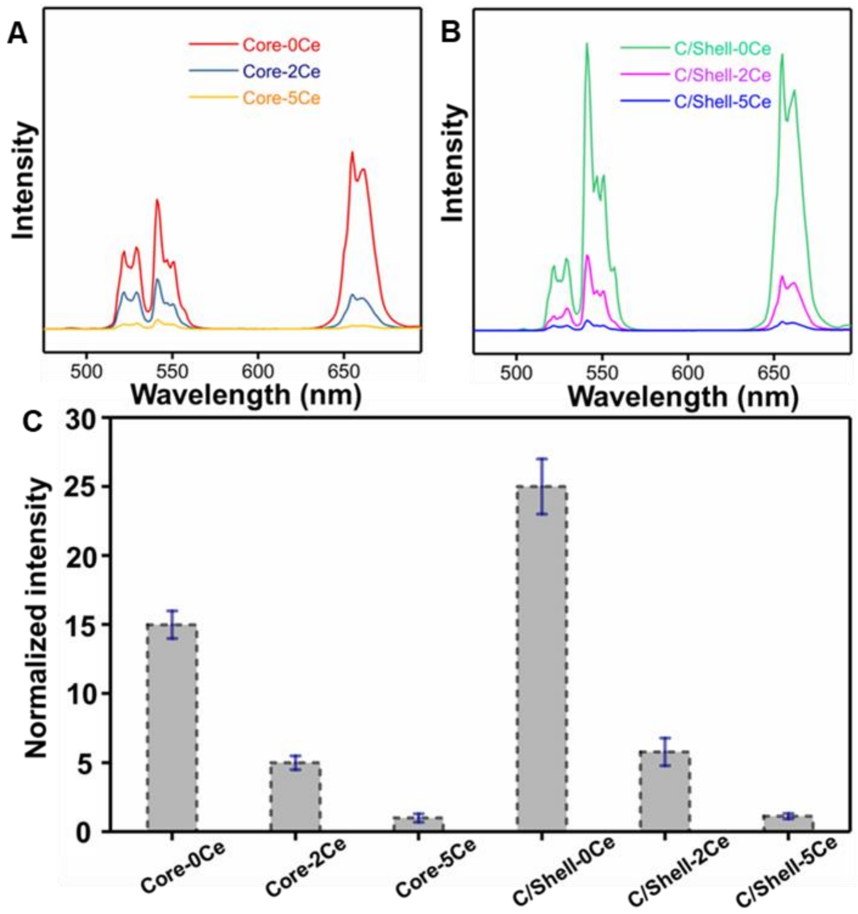

Figure S3. Upconversion emission spectra of the as-prepared Nd-sensitized (A) core and (B) core-shell nanoparticles under 808 nm laser excitation. (C) The normalized upconversion emission intensity of the difference Ce^{3+} doped core and core-shell nanoparticles under 808 nm laser excitation.

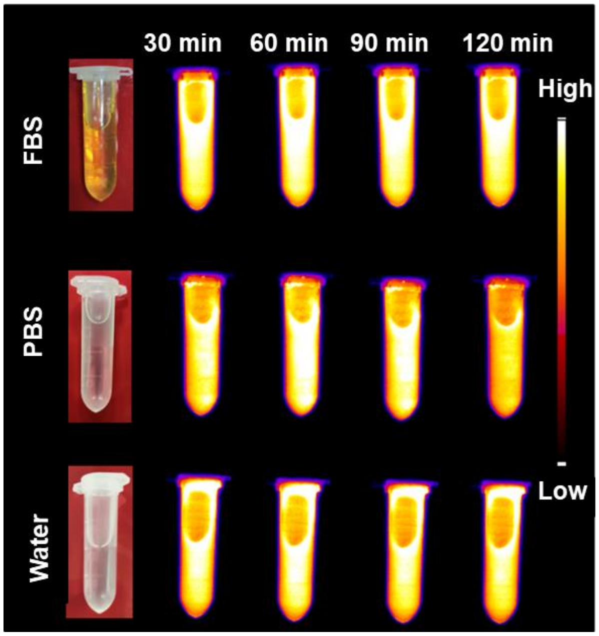

Figure S4. Photostability of the PAA-C/S nanoparticles in FBS, water and PBS under continuous 808 nm laser irradiation.

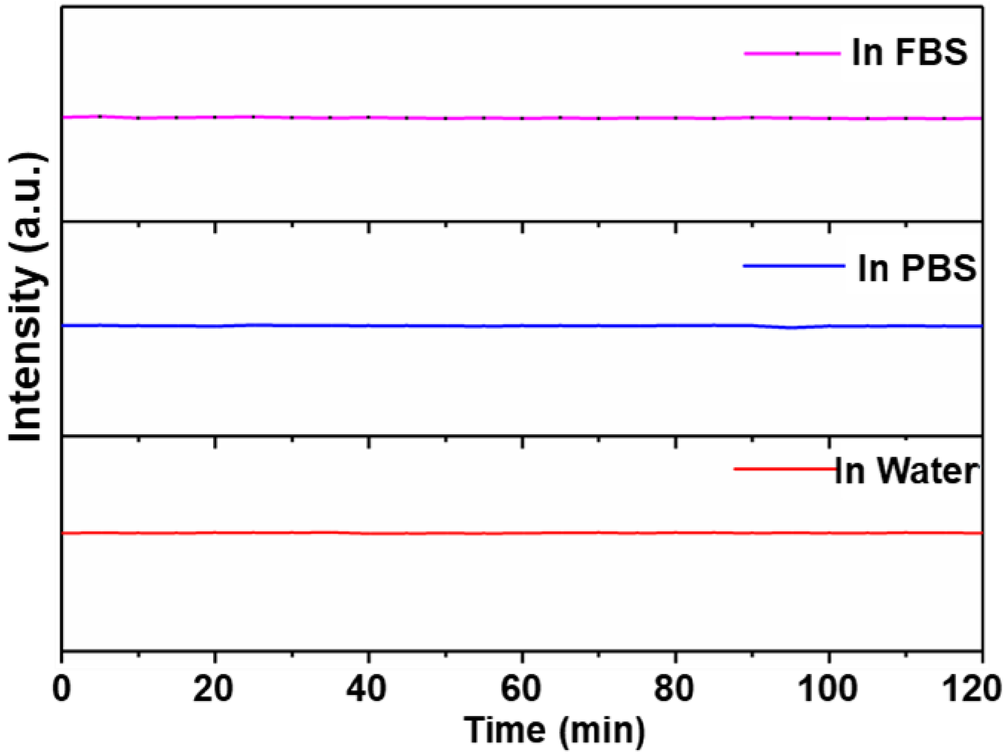

Figure S5. Photostability curves of the PAA-C/S nanoparticles in FBS, PBS and water under continuous 808 nm laser excitation.

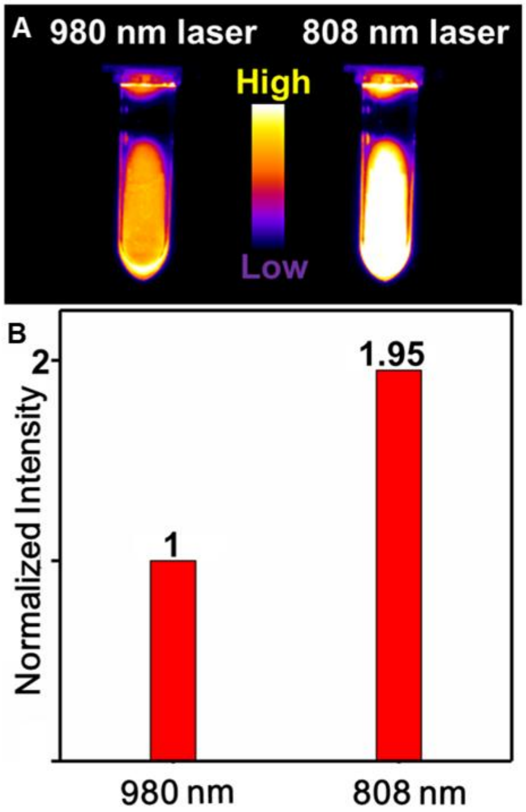

Figure S6. (A) *In vitro* phantom imaging of the $NaYF_4$:Yb/Gd/ 2mol%Ce/Er/Nd@$NaYF_4$:Nd core-shell nanoparticles under 980 nm and 808 nm laser excitation. (B) The corresponding normalized fluorescence intensity of (A).

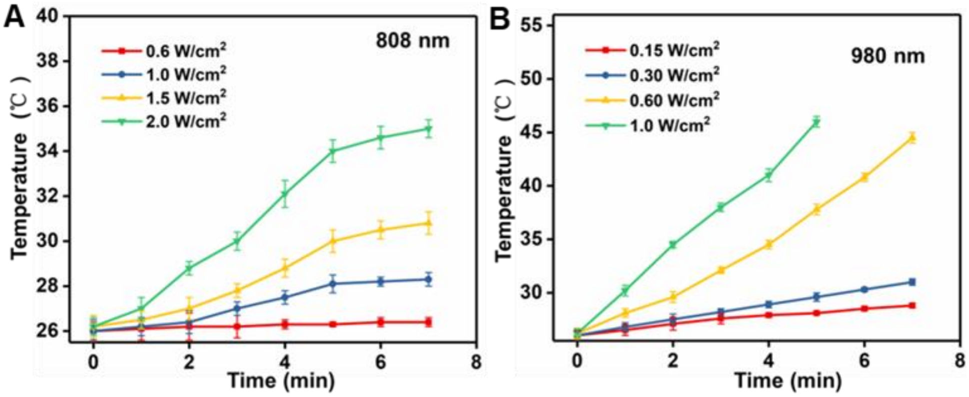

Figure S7. (A) Power-dependent temperature changes of mouse skin upon 808 nm laser excitation. (B) Power-dependent temperature changes of mouse skin upon 980 nm laser excitation.

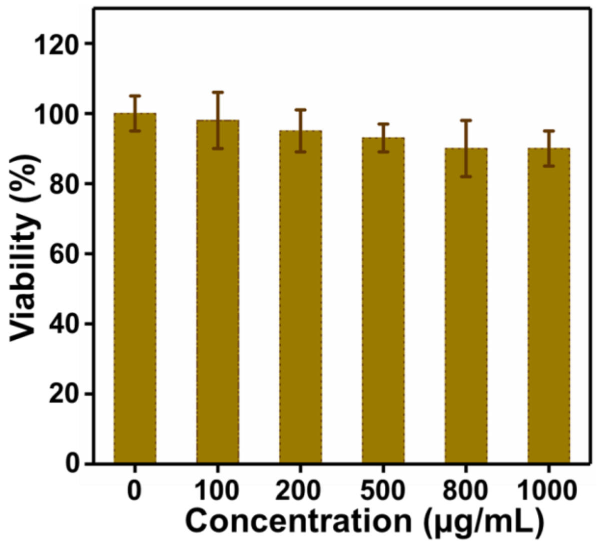

Figure S8. The viability of cell treated with different concentrations (0, 100, 200, 500, 800, 1000 μg/mL) of PAA-C/S nanoprobe by using MTT assay.

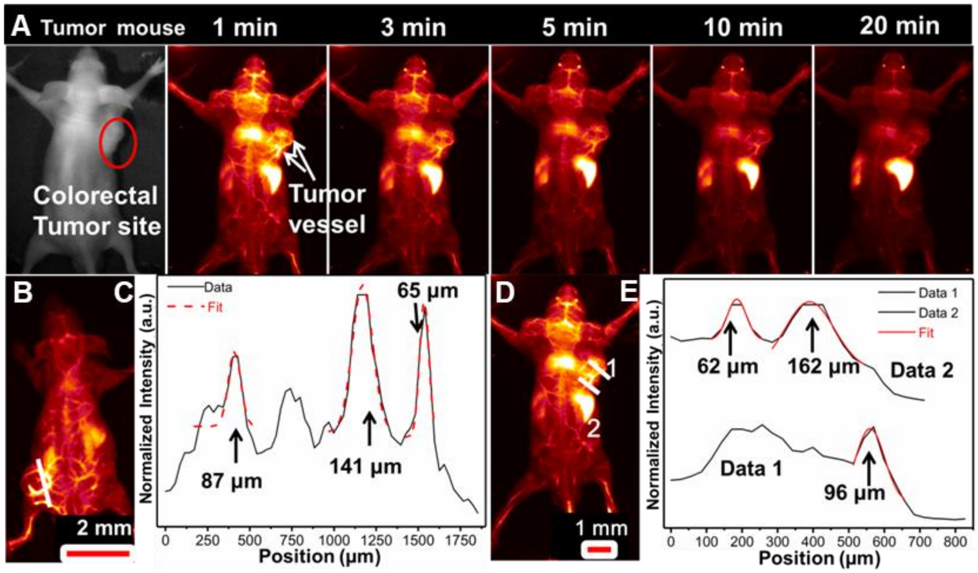

Figure S9. (A) Non-invasive NIR-IIb optical-guided vascular imaging of colorectal tumor bearing mouse. (B) A representative vascular image of the lung tumor-bearing nude mouse in Figure 4B. (C) Cross-sectional intensity profiles, Gaussian functional fitting lines and FWHM values measured along the white line from Figure S9B. (D) A representative tumor vascular imaging of Figure S9A. (E) Cross-sectional intensity profiles, Gaussian functional fitting lines and FWHM values measured along the white lines from Figure S9D.

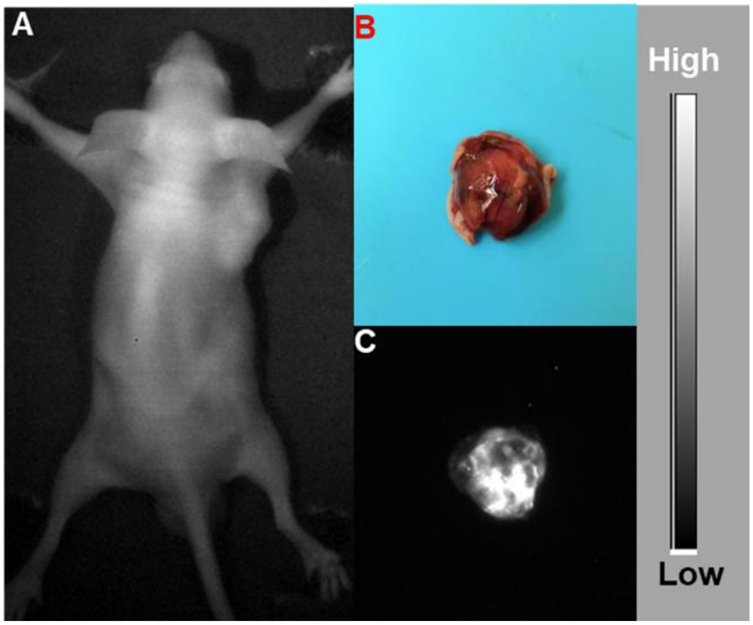

Figure S10. (A) The bright field image of the colorectal tumor-bearing nude mouse. (B) The digital photograph of the dissected colorectal tumor. (C) *Ex vivo* bioimaging of the isolated colorectal tumor resected from the colorectal tumor-bearing nude mouse after intravenous injection with the PAA-C/S nanoprobes 24 h.

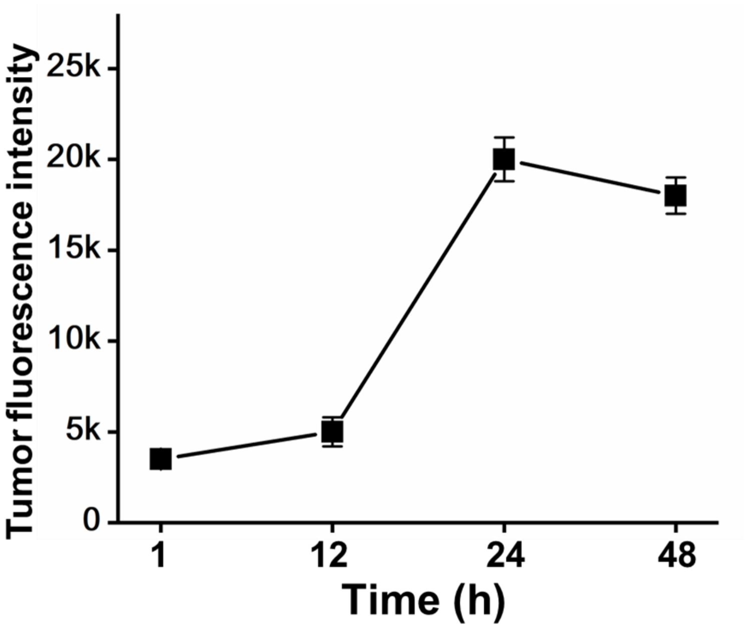

Figure S11. The fluorescence intensity of the tumor site at different time points after intravenous injection.

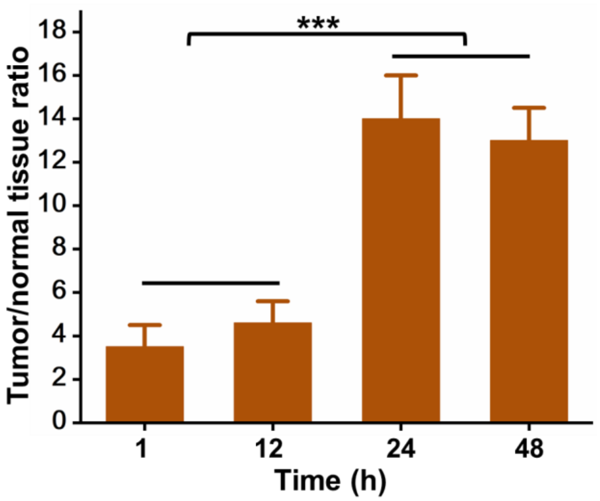

Figure S12. The tumor to normal tissue ratio of the tumor-bearing mice at different time points. Data were presented as mean ± SD (n=3) (***p<0.001).

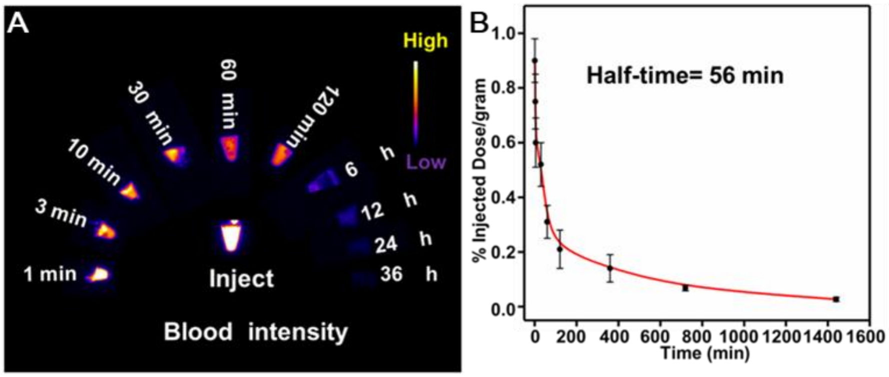

Figure S13. (A) Time-dependent fluorescence images of the blood samples collected at different time points after intravenous injection of PAA-C/S solution. (B) The blood circulation half-life time of the PAA-C/S nanoprobes *in vivo*.

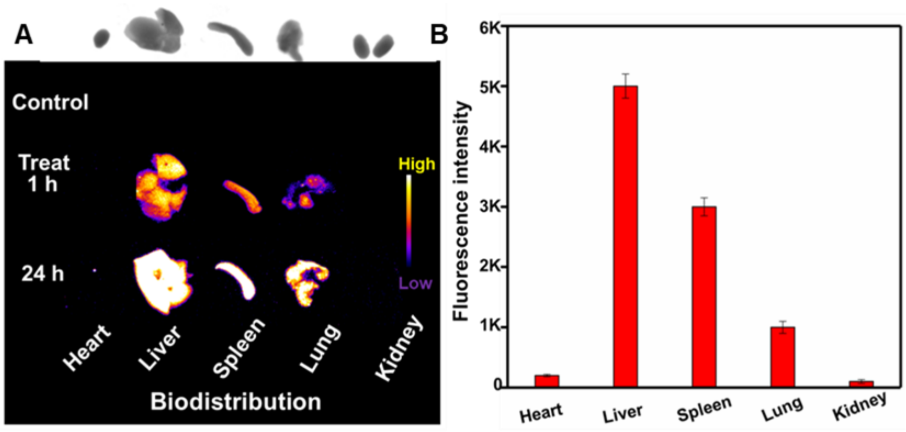

Figure S14. (A) *Ex vivo* NIR-IIb fluorescent images of main organs collected from the control mouse and mice treated with PAA-C/S nanoprobe after 1 h and 24 h post injection under 808 nm laser excitation. (B) Biodistribution of the main organs of PAA-C/S treated mouse after 24 h post injection.

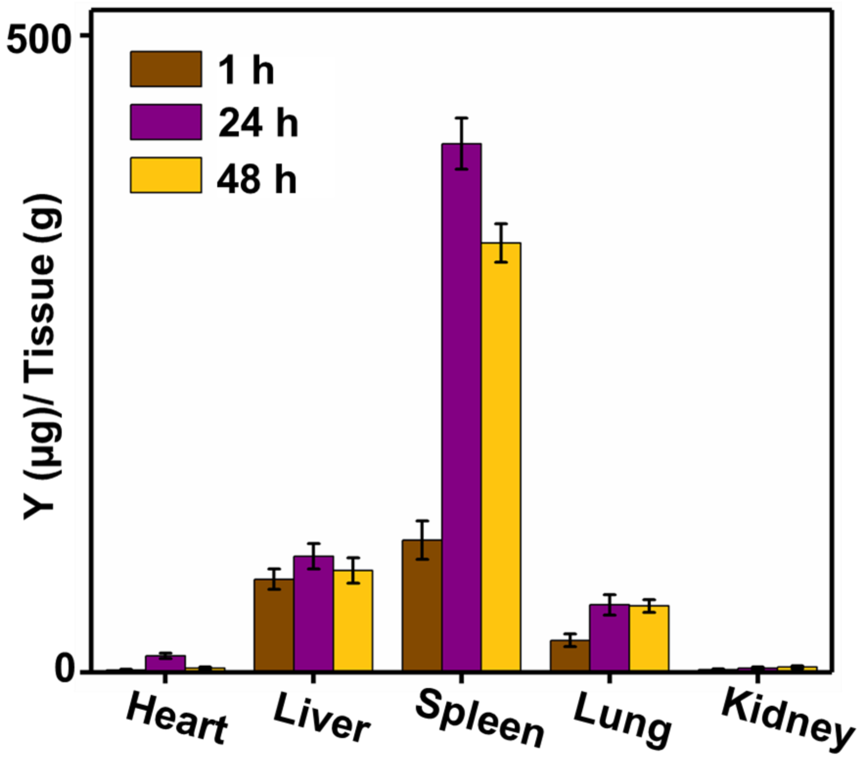

Figure S15. Quantitative biodistribution of Y element in the main organs of mice after 1 h, 24 h, and 48 h injection. The data was measured by ICP-MS.

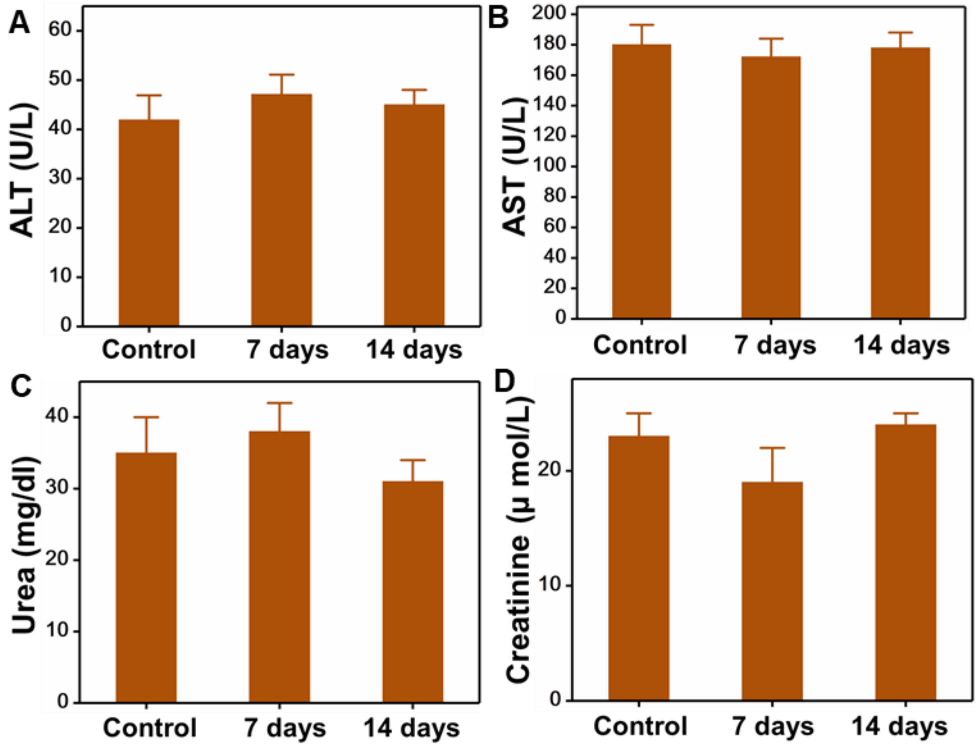

Figure S16. (A)-(D) Blood biochemistry data including aspartate aminotransferase (AST), alanine aminotransferase (ALT), Urea and Creatinine of Kunming mice without injection and at 7, 14 days post-injection of PAA-C/S nanoprobe.

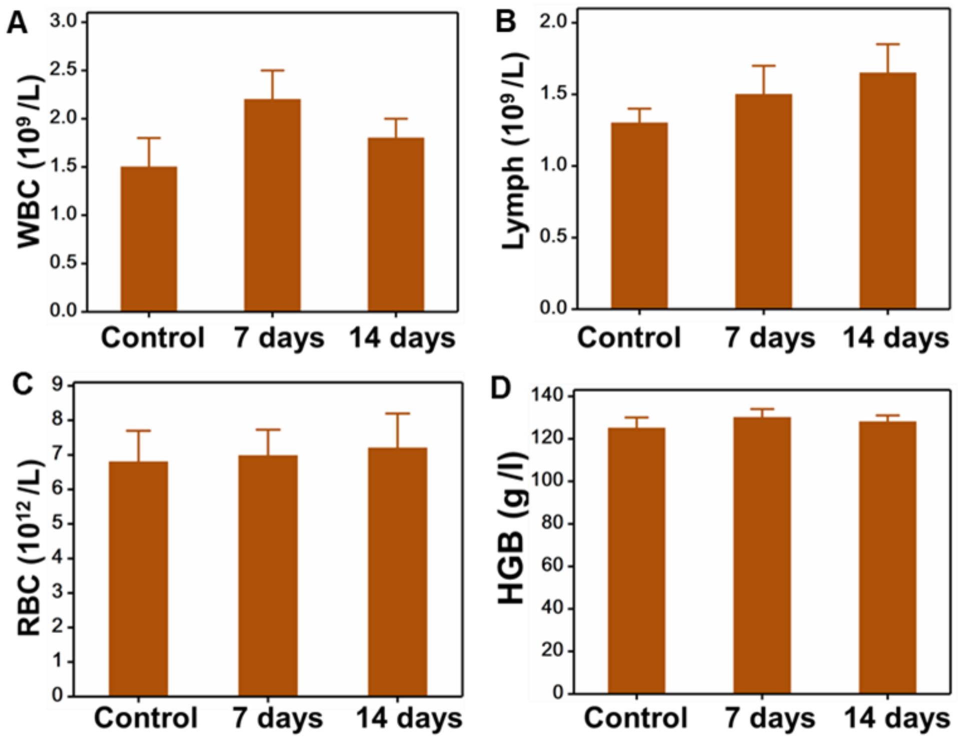

Figure S17. (A)-(D) The blood routine test including white blood cell (WBC), lymph, red blood cell (RBC) and hemoglobin (HGB) of Kunming mice without injection and at 7, 14 days post-injection of PAA-C/S nanoprobe.

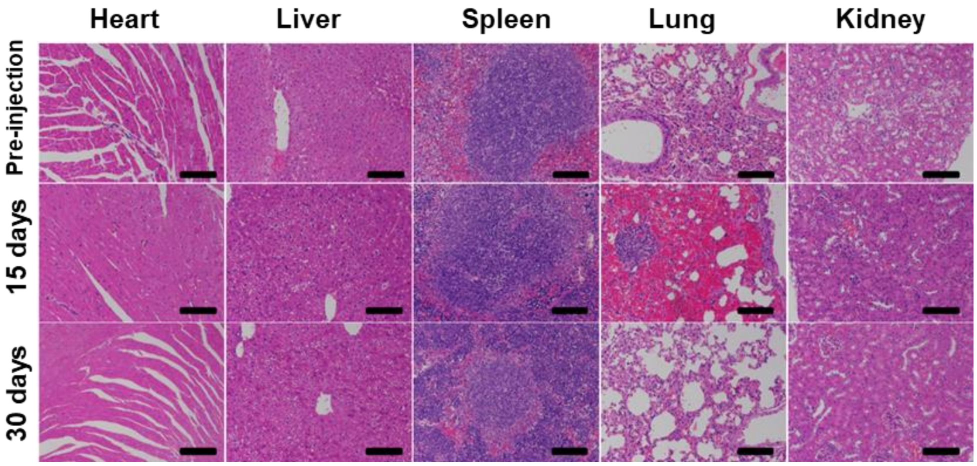

Figure S18. H&E stained images of the main organs collected from the control mouse and mice treated with PAA-C/S nanoparticles for 15 and 30 days.

Supplement Reference:

s1. Xie XJ, Gao NY, Deng RR, Sun Q, Xu QH, Liu XG. Mechanistic investigation of photon upconversion in Nd^{3+}-sensitized core-shell nanoparticles. J Am Chem Soc. 2013; 135: 12608-11..

s2. Wang X, Yakovliev A, Ohulchanskyy TY, Wu L, Zeng SJ, Han XJ, et al. Efficient erbium-sensitized core/shell nanocrystals for short wave infrared bioimaging. Adv Optical Mater. 2018; 6: 1800690.

s3. Xue ZL, Zeng SJ, Hao JH. Non-invasive through-skull brain vascular imaging and small tumor diagnosis based on NIR-II emissive lanthanide nanoprobes beyond 1500 nm. Biomaterials. 2018; 171: 153-63.

s4. Antairs AL, Chen H, Cheng K, Sun Y, Hong GS, Qu CR, et al. A small-molecule dye for NIR-II imaging. Nat Mater. 2016; 15: 235-42.

s5. Zhong YT, Ma ZR, Zhu SJ, Yue JY, Zhang MX, Antaris AL, et al. Boosting the down-shifting luminescence of rare-earth nanocrystals for biological imaging beyond 1500 nm. Nat Commun. 2017; 8: 737.
